# Epitranscriptomic regulation in fasting hearts: implications for cardiac health

**DOI:** 10.1080/15476286.2024.2307732

**Published:** 2024-02-07

**Authors:** Daniel Benak, Kristyna Holzerova, Jaroslav Hrdlicka, Frantisek Kolar, Mark Olsen, Mati Karelson, Marketa Hlavackova

**Affiliations:** a Laboratory of Developmental Cardiology, Institute of Physiology of the Czech Academy of Sciences, Prague, Czech Republic; b Department of Physiology, Faculty of Science, Charles University, Prague, Czech Republic; c Department of Pharmaceutical Sciences, College of Pharmacy-Glendale, Midwestern University, Glendale, Arizona, USA; d Institute of Chemistry, University of Tartu, Tartu, Estonia

**Keywords:** Fasting, heart, epitranscriptomics, m^6^A, m^6^Am, FTO, ALKBH5

## Abstract

Cardiac tolerance to ischaemia can be increased by dietary interventions such as fasting, which is associated with significant changes in myocardial gene expression. Among the possible mechanisms of how gene expression may be altered are epigenetic modifications of RNA – epitranscriptomics. N^6^-methyladenosine (m^6^A) and N^6^,2’-O-dimethyladenosine (m^6^Am) are two of the most prevalent modifications in mRNA. These methylations are reversible and regulated by proteins called writers, erasers, readers, and m^6^A-repelled proteins. We analysed 33 of these epitranscriptomic regulators in rat hearts after cardioprotective 3-day fasting using RT-qPCR, Western blot, and targeted proteomic analysis. We found that the most of these regulators were changed on mRNA or protein levels in fasting hearts, including up-regulation of both demethylases – FTO and ALKBH5. In accordance, decreased methylation (m^6^A+m^6^Am) levels were detected in cardiac total RNA after fasting. We also identified altered methylation levels in *Nox4* and *Hdac1* transcripts, both of which play a role in the cytoprotective action of ketone bodies produced during fasting. Furthermore, we investigated the impact of inhibiting demethylases ALKBH5 and FTO in adult rat primary cardiomyocytes (AVCMs). Our findings indicate that inhibiting these demethylases reduced the hypoxic tolerance of AVCMs isolated from fasting rats. This study showed that the complex epitranscriptomic machinery around m^6^A and m^6^Am modifications is regulated in the fasting hearts and might play an important role in cardiac adaptation to fasting, a well-known cardioprotective intervention.

## Introduction

Ischaemic heart disease is the leading cause of death worldwide [1]. Myocardial ischaemia results in damage to cardiomyocytes which can further lead to impaired heart function. The degree of ischaemic injury, however, depends on the intensity and duration of the ischaemic stimulus and the level of cardiac tolerance to ischaemia [2]. Studies have shown that fasting can attenuate the extent of heart damage caused by myocardial infarction [3,4]. Nevertheless, the molecular mechanisms responsible for this cardioprotective phenotype are not yet fully resolved.

Epitranscriptomic modifications are dynamic changes to the chemical composition of RNA that have the potential to alter its stability or function [5]. N^6^-methyladenosine (m^6^A) and N^6^,2’-O-dimethyladenosine (m^6^Am) are among the most common modifications [6–8]. These methylations profoundly affect gene expression regulation and cellular physiology and pathophysiology. Proteins called writers (methylation deposition), erasers (methylation removal), readers (binding of modified RNA), and also m^6^A-repelled proteins (binding of unmodified RNA) mediate the biological effects of these modifications [9,10] (details in Table 1).

**Table 1.** The m^6^A and m^6^Am regulators.

Protein	Protein name	Function	Modification
**METTL3**	Methyltransferase-like 3	Writers	m^6^A
METTL14	Methyltransferase-like 14		m^6^A
WTAP	Willms‘ tumour 1-associating protein		m^6^A
METTL5	Methyltransferase-like 5		m^6^A
METTL16	Methyltransferase-like 16		m^6^A
ZCCHC4	Zinc finger CCHC-type containing 4		m^6^A
**PCIF1**	Phosphorylated CTD interacting factor 1		m^6^Am
**METTL4**	Methyltransferase-like 4		m^6^Am
**FTO**	Fat mass and obesity-associated protein	Erasers	m^6^Am, m^6^A
**ALKBH5**	AlkB family member 5		m^6^A
**YTHDF1**	YTH domain-containing family protein 1	Readers	m^6^A
**YTHDF2**	YTH domain-containing family protein 2		m^6^A
**YTHDF3**	YTH domain-containing family protein 3		m^6^A
**YTHDC1**	YTH domain-containing protein 1		m^6^A
**YTHDC2**	YTH domain-containing protein 2		m^6^A
eIF3a	Eukaryotic initiation factor 3a		m^6^A
eIF3c	Eukaryotic initiation factor 3c		m^6^A
eIF3g	Eukaryotic initiation factor 3g		m^6^A
HNRNPA2B1	Heterogeneous nuclear ribonucleoprotein A2/B1		m^6^A
HNRNPC	Heterogeneous nuclear ribonucleoprotein C		m^6^A
HNRNPD	Heterogeneous nuclear ribonucleoprotein D		m^6^A
RBMX (HNRNPG)	RNA-binding motif protein, X chromosome		m^6^A
IGF2BP1	Insulin-like growth factor 2 mRNA binding protein 1		m^6^A
IGF2BP2	Insulin-like growth factor 2 mRNA binding protein 2		m^6^A
IGF2BP3	Insulin-like growth factor 2 mRNA binding protein 3		m^6^A
FMR1	Fragile X messenger ribonucleoprotein 1		m^6^A
PRRC2A	Proline rich-coil 2A		m^6^A
G3BP1	G3BP stress granule assembly factor 1	m^6^A-repelled proteins	m^6^A
G3BP2	G3BP stress granule assembly factor 2		m^6^A
ELAVL1 (HUR)	ELAV-like protein 1		m^6^A
USP10	Ubiquitin specific peptidase 10		m^6^A
CAPRIN1	Cell cycle associated protein 1		m^6^A
RBM42	RNA binding motif protein 42		m^6^A

The key regulators are in bold.

Epitranscriptomic regulations play a wide range of roles in cardiovascular health and disease [5,11–14]. For instance, the levels of m^6^A and its writer METTL3 are up-regulated in hearts after ischaemia-reperfusion (I/R) injury [15]. Changes in m^6^A-RNA methylation are also associated with heart failure [16,17]. Notably, the epitranscriptomic regulators also play a role in cardioprotection [18–21]. Particularly, the well-known eraser fat mass and obesity-associated protein (FTO) is mostly associated with beneficial effects on the heart [18,19]. However, only one study has focused on fasting animal hearts so far. This study described that intermittent fasting (IF) improved high-fat diet-induced cardiomyopathy via an FTO-associated decrease in m^6^A methylation [22]. These limited data suggest that epitranscriptomics might represent a crucial layer of gene regulation in fasting hearts. Thus, investigating the potential role of epitranscriptomic modifications and their regulators in the induction of cardioprotection during fasting is of great importance.

In this study, we performed a detailed analysis of 33 m^6^A and m^6^Am regulators in the hearts of rats subjected to 3-day fasting. We showed that most of the epitranscriptomic regulators were affected by fasting, including up-regulation of demethylases FTO and ALKBH5, and that RNA methylation levels were decreased in the fasting hearts. At the same time, some of the transcripts of genes participating in possible protective pathways were up-methylated. Moreover, we studied the inhibition of ALKBH5 and FTO in rat adult left ventricular cardiomyocytes (AVCMs) and found that inhibition of both demethylases decreased the hypoxic tolerance of AVCMs isolated from fasting rats. Our data suggest that epitranscriptomics might play an essential role in the molecular adaptation of the heart to fasting, a promising cardioprotective intervention.

## Materials and methods

### Animals and experimental protocol

Adult (12-week-old) male Wistar rats were divided into two groups. The experimental group was kept without food for 3 days but had free access to water [4]. The control group was fed *ad libitum*. All animals were housed in a controlled environment (23°C; 12 h light–dark cycle; light from 6:00 AM). The study followed the Guide for the Care and Use of Laboratory Animals (published by the National Academy of Science, National Academy Press, Washington, DC, USA). Experimental protocols were approved by the Animal Care and Use Committee of the Institute of Physiology, The Czech Academy of Sciences.

### Blood glucose and haematocrit levels

Glucose levels in the tail blood were measured before the onset of fasting and after each day of fasting using a glucometer. Haematocrit was determined at the end of fasting by the capillary micromethod.

### Echocardiography

The geometry and function of the left ventricle (LV) were assessed by echocardiography after 3 days of fasting using GE Vivid 7 Dimension (GE Vingmed Ultrasound, Horten, Norway) with a 12 MHz linear matrix probe M12L [23]. Animals were anesthetized with 2% isoflurane (Forane, Abbott Laboratories, Queenborough, United Kingdom) mixed with room air, placed on a heating pad and their rectal temperature was maintained between 35.5 and 37.5°C. Basic 2-D and M-modes were recorded in both the long and short axes. Heart rate (HR) and the following parameters of LV geometry were assessed: end-diastolic and end-systolic LV cavity diameter (LVDd, LVDs), anterior wall thickness (AWTd, AWTs), and posterior wall thickness (PWTd, PWTs). Fractional shortening (FS), relative wall thickness (RWT), and cardiac index (CI) were derived as follows: FS = 100*[(LVDd-LVDs)/LVDd]; RWT = 100*[(AWTd+PWTd)/LVDd]; CI = [(π/3)*LVDd^3^]-[(π/3)*LVDs^3^]*HR/BW.

### Heart catheterization

After the echocardiographic examination, the anesthetized rats were subjected to LV catheterization through the right carotid artery using the SPR-407 microtip pressure catheter as described previously [24]. Data were acquired using MPVS 300 (Millar, Houston, Texas, USA) and PowerLab 8/30 (ADInstruments, Oxford, UK). End-diastolic pressure (Ped), end-systolic pressure (Pes), developed pressure (Pdev), and peak rate of pressure development and decline (+(dP/dt)_max_, -(dP/dt)_max_, respectively) were assessed from 5 consecutive pressure cycles using LabChart Pro (ADInstruments, Oxford, UK).

### Collection of tissue samples

Immediately after the fasting period, the rats were killed by cervical dislocation. The hearts were rapidly excised, washed in a cold (0°C) saline, and dissected into RV, LV, and the septum [25]. All collected tissue segments were weighed, frozen, and stored in liquid nitrogen until use. The heart weight was normalized to tibia length.

### RNA isolation, cDNA synthesis, and RT-qPCR analysis

Total RNA was extracted from each LV sample using RNAzol® RT according to the manufacturer’s instructions. The concentration of total RNA was measured on NanoDrop 1000 (Thermo Fisher Scientific, USA). One µg of total RNA was used to synthesize first-strand cDNA using RevertAid H Minus First Strand cDNA Synthesis Kit (Thermo Fisher Scientific, USA) and random primers according to the manufacturer’s protocol. RT-qPCR was performed in 20 µl reaction volume on a LightCycler® 480 (Roche Diagnostics, Switzerland) using TaqMan Gene Expression Assays (Tab. S1; Thermo Fisher Scientific, USA) and 5× HOT FIREPol Probe qPCR Mix Plus (NO ROX) (Solis Biodyne, Estonia) according to the manufacturer’s instructions with the following temperature profile: initial enzyme activation (15 min at 95°C) followed by 45 cycles of amplification (15 s at 95°C, 1 min at 60°C) [26]. For proper normalization [27], suitable reference genes were assessed. In total, six reference genes were evaluated: hypoxanthine phosphoribosyltransferase 1 (*Hprt1*), nucleoporin-like 2 (*Nupl2*), succinate dehydrogenase complex flavoprotein subunit A (*Sdha*), translocase of outer mitochondrial membrane 22 (*Tomm22*), DNA topoisomerase I (*Top1*), and tyrosin-3-monooxygenase/tryptophan 5 monooxygenase activation protein zeta (*Ywhaz*). *Ywhaz* and *Top1* were selected as the most stable of these genes and were used for normalization. Data were analysed by instructions from qPCR courses performed by TATAA Biocenter (http://www.tataa.com/courses/).

### SDS-PAGE and Western blot analysis

Tissue homogenization, protein separation, and immunodetection were performed as described earlier [13]. Each frozen LV was pulverized in liquid nitrogen to a fine powder followed by Potter-Elvehjem homogenization in eight volumes of homogenization buffer [12.5 mM TRIS, 2.5 mM EGTA, 250 mM sucrose, 6 mM 2-mercaptoethanol, protease inhibitor cocktail (Roche) and phosphatase inhibitor cocktail (Roche, Switzerland), pH 7.4]. The protein concentration in homogenates was measured by the Bradford method (Bio-Rad, USA). The LV homogenates were subjected to SDS electrophoresis on 10% polyacrylamide gels (Mini-PROTEAN TetraCell, Bio-Rad, USA) and electrotransferred onto PVDF membranes (0.2 μm pore size, Bio-Rad). Subsequently, membranes were blocked with 5% blotting-grade blocker (Bio-Rad, USA) in PBS containing Tween 20 (1%) for 1 h and incubated with appropriate primary and secondary antibodies (diluted in 1% blotting-grade blocker and 1% Tween 20 in PBS): anti-FTO (Abcam, ab92821, 1:1,000, overnight), anti-ALKBH5 (Abcam, ab195377, 1:1,400, overnight), anti-METTL3 (Abcam, ab195352, 1:1,000, overnight), anti-PCIF1 (Invitrogen, PA5–110081, 1:1,400, overnight), anti-METTL4 (Invitrogen, PA5–97202, 1:1,400, overnight), anti-YTHDF1 (Abcam, ab157542, 1:1,400, overnight), anti-YTHDF2 (Invitrogen, PA5–70853, 1:1,400, overnight), anti-YTHDF3 (Sigma-Aldrich, SAB21022736, 1:1,400, overnight), anti-YTHDC1 (Abcam, ab220159, 1:1400, overnight), anti-YTHDC2 (Abcam, ab220160, 1:1,400, overnight), anti-mouse secondary antibody (ThermoFisher 31432, 1:10,000, 1 h) and anti-rabbit secondary antibody (Bio-Rad, 170–6515, 1:10,000, 1 h). The same amount of protein was loaded on the gels for all samples. The results were recalculated to the total protein amount gained by Ponceau S staining [28]. Each sample was analysed at least three times. The membranes were visualized by enhanced chemiluminescence (ECL) substrates (RNAzol® West Dura Extended Duration Substrate or LightCycler® West Femto Maximum Sensitivity Substrate, Thermo Scientific) using a SuperSignal™ system (Bio-Rad, Hercules, USA). Quantification of the results was performed using ImageJ software.

### Targeted proteomic analysis

Samples were dissolved in 25 µl loading buffer (0.05% TFA, 2% acetonitrile) and firstly analysed using data-independent-acquisition (DIA). For targeted analysis, samples were spiked with a mix of 72 isotopically labelled peptides containing C-terminal 15N and 13C-labelled arginine and lysine residues (JPT Peptide Technologies GmbH, Berlin, Germany) to a concentration corresponding to 1 fmol/peptide on a column. Before internal standard (IS) spiking, samples were diluted to an estimated amount of 1 µg of the total peptide on a column. Due to detection limits above 1 fmol/peptide on a column for some internal standards, samples were re-spiked to 40 fmol IS peptides on a column for a second injection.

For LC-MS analysis, an Ultimate 3000 liquid chromatograph coupled to an Orbitrap Exploris 480 mass spectrometer equipped with FAIMS was used. Peptides were loaded onto a PepMap Neo 0.5 cm x 300 µm i.D., 5 µm C18, 100 A trap column (Thermo Fisher Scientific) for 2 min at 17.5 µl/min. Separation and subsequent ion spray ionization were performed on a 50 cm x 75 µm i.D. Easy-Spray column with 2 µm C18 particles and 100 A pore size. A solvent gradient from 97% mobile phase A (0.1% FA in H_2_O) to 35% mobile phase B (0.1% formic acid in 80% acetonitrile) for 60 min was used for targeted acquisition and 120 min for DIA analysis. The spray voltage was set to 2,000 V for all runs. FAIMS was run in standard resolution mode for DIA runs for parallel reaction monitoring analysis (PRM) and low-resolution mode (inner electrode temp.: 100°C, outer electrode temp.: 80°C). Compensation voltage was fixed to -45 V for DIA runs but individually optimized for each of the 72 peptides for PRM analysis (CVs used: -35, -40, -45, -50, -60, -70). Analysis in data-independent mode was performed with the following settings: MS1 resolution of 60,000 FWHM with a scan range between *m*/*z* 350 and 1,500; injection time of 100 ms and an AGC of 300% (3 × 10^6^). For peptide spectrum generation 2 × 38 staggered MS2 scans with an isolation width of *m/z* 16 including precursors from *m/z* 400 to 1,000, without overlap were defined. The corresponding instrument settings were 27% HCD collision energy 30,000 FWHM resolution, 55 ms ion injection time, and an AGC target of 1,000% (1 × 10^6^). The targeted analysis consisted of PRM scans for light and heavy precursors with isolation widths of *m/z* 1.6, a resolution of 60,000 FWHM; 118 ms ion injection time, an AGC target of 1 × 10^5^, and HCD collision energy set to 27%.

Acquired raw files from DIA runs were analysed in Spectronaut. PRM data were analysed in Skyline-daily. Transition areas were integrated and normalized for relative quantification to isotopically labelled heavy internal standard peptides. Normalized ratios on both peptide and protein levels were exported to Excel and relative changes between groups were computed.

### Untargeted lipidomics and metabolomics

Plasma samples were extracted using a biphasic solvent system of cold methanol, methyl *tert*-butyl ether, and water [29]. Then, a multiplatform LC-MS-based approach [30] was used for metabolomic and lipidomic profiling, with details summarized in Supplementary Materials.

### m^6^A/m quantification in total RNA from left ventricles

The m^6^A/m (m^6^A + m^6^Am) levels in the total RNA samples were detected by the EpiQuik m6A RNA Methylation Quantification Kit (Epigentek, Farmingdale, USA) according to the manufacturer’s instructions. For each analysis, 300 ng of RNA was used. The absorbance was read on a microplate reader Synergy™ HT Multi-Detection Microplate Reader (BioTek, Winooski, USA) at 450 nm. The estimation of the m^6^A/m percentage in RNA was done using the formula: m^6^A/m (%) = [(sample OD-negative control OD)/Slope]*100%. The results of this assay were described as m^6^A/m levels because this method does not differentiate between m^6^A and m^6^Am modifications [14].

### m^6^A RNA immunoprecipitation (MeRIP)

The immunoprecipitation of m^6^A/m-modified RNA was done using Magna MeRIP^TM^ m^6^A Kit (Merck Millipore, Burlington, USA) following the manufacturer’s instructions. Briefly, 80 μg of total RNA isolated from LVs was fragmented at 94°C for 5 min following incubation with magnetic beads at 4°C for 2 h. After that, samples were eluted with elution buffer containing N^6^-Methyladenosine 5’-monophosphate sodium salt. Eluted RNA was purified using PureLink^TM^ RNA Mini Kit (Thermo Fisher Scientific, USA). Genes potentially participating in cardioprotection induced by fasting [31] – NFE2 like BZIP transcription factor 2 (*Nfe2l2*), Sirtuin 1 (*Sirt1*), Sirtuin 3 (*Sirt3*), Protein kinase AMP-activated catalytic subunit alpha 2 (*Prkaa2*), RELA proto-oncogene, NF-KB Subunit (*Rela*), NADPH oxidase 4 (*Nox4*), Histone deacetylase 1 (*Hdac1*), Forkhead box O3 (*Foxo3*), Hypoxia-inducible factor 1 subunit alpha (*Hif1a*) – were selected for analysis of MeRIPed RNA, which was performed by RT-qPCR as described above. TaqMan Gene Expression Assays used for this analysis are listed in the supplements (Tab. S2).

### AVCM isolation and culture

The rat AVCMs were isolated from 12-week-old male Wistar rats as described previously [32] with slight adjustments. The rats were heparinized (5,000 U/kg, i.p.), anesthetized by intraperitoneal injection of pentobarbital (60 mg/kg), and killed by cervical dislocation. The hearts were rapidly excised and perfused for 10 min with Ca^2+^-free buffer containing 10 mM KCl, 1.2 mM K_2_HPO_4_, 90 mM NaCl, 5 mM MgSO_4_, 15 mM NaHCO_3_, 20 mM glucose, and 30 mM taurine (pH 7.4) at 37°C. The perfusion medium was then switched to Ca^2+^-free buffer containing collagenase Type 2 (8,000 U; Worthington, Lakewood, USA), bovine serum albumin (0.2%), and Ca^2+^ (50 μM). All solutions were gassed with 95% O_2_ and 5% CO_2_ for 30 min before use. After 60 min of digestion, the LV was minced, and cardiomyocytes were isolated by sedimentation in a gradually increasing Ca^2+^ concentration buffer until a final concentration of 1.2 mM. Finally, the myocytes isolated from the LV were gently resuspended in a cell culture medium-M199 (M199, containing 5% FBS, 100 U/ml penicillin, and 100 μg/ml streptomycin) and transferred to laminin-coated culture dishes and let in a CO_2_ incubator (95% air, 5% CO_2_, 37°C) for 2 h to attach.

### FTO and ALKBH5 inhibitors

A pharmacological inhibitor of FTO (FTOi; MO-I-500 [33]) was dissolved in DMSO as 1 mM stocks and stored at −20°C in small aliquots. A pharmacological inhibitor of ALKBH5 (ALKBH5i; compound 3 [34]) was dissolved in DMSO as 50 mM stocks and stored at 5°C in small aliquots. DMSO (0.1% final concentration) was also used as vehicle control.

The dose-response of the viability of AVCMs to FTOi and ALKBH5i was tested in this study to select the appropriate concentration of inhibitors (Fig. S1). The viability of AVCMs was determined after 24 h incubation with FTOi (0.5, 1, 2.5, 5, 10, 50 μM) or ALKBH5i (10, 50, 75, 100, 150 μM) using SYTOX Green nucleic acid stain (S7020) (Invitrogen-Molecular Probes, Eugene, USA). Based on these results, the 1 μM (FTOi) and 50 μM (ALKBH5i) concentrations, which did not significantly affect the number of surviving cells during 24 h incubation, have been chosen for the following experiments.

## Hypoxic tolerance of cardiomyocytes

AVCMs isolated either from control or fasting rats were incubated in hypoxic chamber Xvivo System X3 (BioSpherix, USA) under hypoxic conditions (1% O_2_; 5% CO_2_; 37°C) for 24 h in M199 medium containing 1 μM FTOi or 50 μM ALKBH5i or 0.01% DMSO. Control AVCMs were incubated under normoxic conditions (95% air, 5% CO_2_, 37°C) with or without 1 μM FTOi or 50 μM ALKBH5i.

The percentage of living cells compared to the untreated normoxic cells was determined using the SYTOX Green nucleic acid stain (S7020) (Invitrogen-Molecular Probes, Eugene, USA) at the beginning of the experiments (after stabilization), after 24 h of treatment, and finally after incubation with 8% Triton X-100 [35]. The overall fluorescence of the cells is inversely related to the intactness of the cell membranes. The fluorescence of SYTOX Green was measured at 490 nm excitation and 520 nm emission wavelengths in 96-well laminin-coated plates (at 8,000 cells per well) using the Synergy™ HT Multi-Detection Microplate Reader (BioTek, Winooski, USA).

## Statistical analyses

All experiments included 6–10 biological replicates per group, except for MeRIP analysis (*n* = 3). Statistical analyses were performed using GraphPad Prism 8 (GraphPad Software, San Diego, USA). Data were expressed as means ± SD. Unpaired two-sided Student’s t-test or one-way ANOVA followed by Tukey’s multiple comparisons test were used to assess statistical significance when comparing two or more groups, respectively. Differences with a p-value ≤0.05 were considered statistically significant.

## Results

### Characteristics of the fasting model

The average body weight (BW) of control (421 g) and fasting (426 g) rats did not differ before the onset of the experimental protocol. On average, after 3 days of fasting, the rats lost 71 g (17%) of BW, while control rats gained 4 g (+3%) of BW by the same period. The hearts of fasting rats were smaller by 16% compared to control rats after normalization to tibia length (Table 2).

**Table 2.** Characteristics of the fasting model.

	Control rats	Fasting rats		
BW change (%)	+3 ± 2.16	-17 ± 2.22*		
HW/Tibia (%)	25.5 ± 1.50	22 ± 1.47***		
Hematocrit (%)	40.3 ± 4.49	45.6 ± 2.97*		
Glycemia (mmol/l)	6.2 ± 0.43	3.9 ± 0.77****		
AWTd (mm)	1.96 ± 0.13	1.85 ± 0.15		
AWTs (mm)	2.81 ± 0.09	2.66 ± 0.23		
PWTd (mm)	1.83 ± 0.13	1.90 ± 0.19		
PWTs (mm)	2.71 ± 0.14	2.67 ± 0.24		
RWT (%)	49.88 ± 5.59	51.41 ± 3.67		
LVDd (mm)	7.63 ± 0.42	7.23 ± 0.17*		
LVDs (mm)	4.64 ± 0.27	4.72 ± 0.22		
FS (%)	39.9 ± 1.8	35.1 ± 3.4*		
HR (bpm)	350 ± 19.9	327 ± 22*		
CI (ml/min/kg)	306 ± 62	276 ± 33		
Pes (mmHg)	86.63 ± 5.69	89.38 ± 4.34		
Ped (mmHg)	4.00 ± 1.38	4.28 ± 2.25		
Pdev (mmHg)	82.63 ± 4.88	85.10 ± 4.47		
+(dP/dt)_max_ (mmHg/s)	7,008 ± 529	5,453 ± 417*		
-(dP/dt)_max_ (mmHg/s)	-7,080 ± 529	-6,592 ± 616		

Values are means ± SD; *n* = 8–10; *p < 0.01; ***p < 0.001, ****p < 0.0001. AWTd – end-diastolic anterior wall thickness; AWTs – end-systolic anterior wall thickness; bpm – beats per minute; BW – body weight; CI – cardiac index; FS – fractional shortening; HR – heart rate; HW – heart weight; LVDd – end-diastolic LV diameter; LVDs – end-systolic LV diameter; Ped – end-diastolic pressure; Pes – end-systolic pressure; Pdev – developed pressure; PWTd – end-diastolic posterior wall thickness; PWTs – end-systolic posterior wall thickness; RWT – relative wall thickness; +(dP/dt)max – peak rate of pressure development; -(dP/dt)max – peak rate of pressure decline.

**Figure 1.**
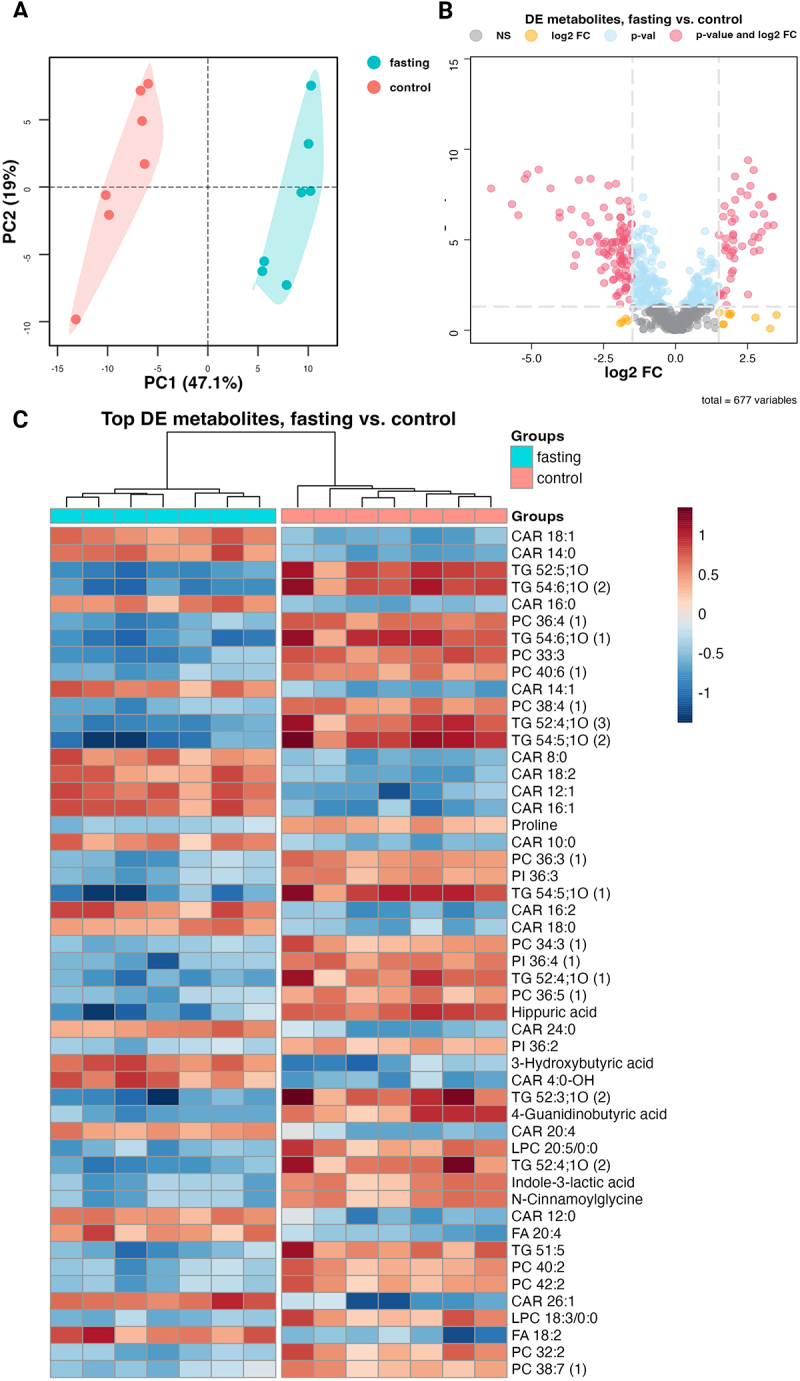
Effect of fasting on plasma metabolites measured by a multiplatform LC-MS-based approach (A) PCA (principal component analysis) showing a clear separation between fasting and control rat plasma samples, suggesting distinct metabolomic profiles associated with the fasting state (B) volcano plot of all (677) metabolites indicating differential levels of metabolites in plasma samples of fasting and control rats (C) heat map of the 50 most significantly affected metabolites; *n* = 7.

The haematocrit in fasting rats (45.6%) was significantly higher than in controls (40.3%) The glycaemia dropped from 6.2 mmol/l to 3.5, 3.7, and 3.9 mmol/l after the first, second, and third day, respectively (Table 2). In total, 677 metabolites were detected in plasma samples. Fasting rats exhibited 171 down-regulated and 79 up-regulated metabolites (Figure 1). The most down-regulated included triacylglycerols, proline, hippuric acid, phosphatidylinositols, phosphatidylcholines, and lysophosphatidylcholines. The most up-regulated included free fatty acids, acylcarnitines, and 3-hydroxybutyric acid. These results demonstrated a shift in metabolism towards lipids and the generation of ketone bodies typical for fasting [4,36].

Echocardiographic assessment of LV geometry and function (Table 2) did not show differences in wall thicknesses in control and fasting rats. LVDd was decreased in fasting rats and LVDs did not differ between the two groups. FS and HR were lower in fasting rats. CI, a parameter describing the cardiac output corrected to the BW, did not differ significantly between the two groups.

LV catheterization (Table 2) showed differences between experimental groups in neither Pes nor Ped. Developed pressure was not altered by fasting. While +(dP/dt)_max_ decreased in fasting rats, -(dP/dt)_max_ was not significantly affected by fasting.

While the observed metabolic and physiological changes, including weight loss and altered plasma metabolites, are consistent with a prolonged fasting response, it is important to note that many cardiac function parameters remained unaffected. This highlights a selective impact of fasting on different body systems, indicating a complex adaptive response to nutritional stress.

### Effect of fasting on levels of m^6^A and m^6^Am regulatory proteins and transcripts in the left ventricles

**Figure 2.**
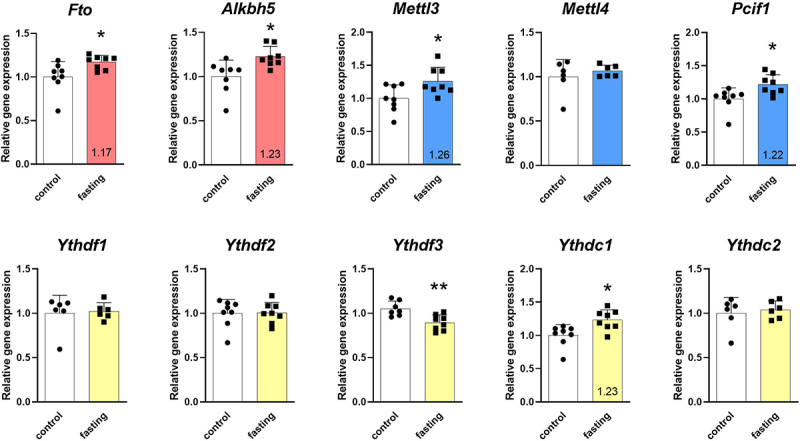
Effect of 3-day fasting on gene expressions of m^6^A and m^6^Am regulators in the left ventricle assessed by RT-qPCR. Writers are displayed in blue, erasers in red, and readers in yellow. The average of the control values is set to 1. Values are means ± SD; *n* = 6–8; *p < 0.05; **p < 0.01 (t-test). *Alkbh5* – AlkB family member 5; *Fto* – fat mass and obesity-associated; *Mettl3* – methyltransferase-like 3; *Mettl4* – methyltransferase-like 4; *Pcif1* – phosphorylated CTD interacting factor 1; *Ythdf1–3* – YTH domain-containing family protein 1–3; *Ythdc1–2* – YTH domain-containing protein 1–2.

Firstly, the effect of fasting on the main m^6^A and m^6^Am machinery gene expression was evaluated in LV samples of fasting and control rats by RT-qPCR (Figure 2). Both erasers were up-regulated, *Fto* by 17%, and *Alkbh5* by 23%. Two out of three writers were also up-regulated, *Mettl3* by 26%, and *Pcif1* by 22%. Regarding readers, only two were affected by fasting. *Ythdf3* levels were decreased by 15% and *Ythdc1* levels were increased by 23%. Other transcript levels (*Mettl4*, *Ythdf1*, *Ythdf2*, *Ythdc2*) were stable in the LVs of starving rats.

**Figure 3.**
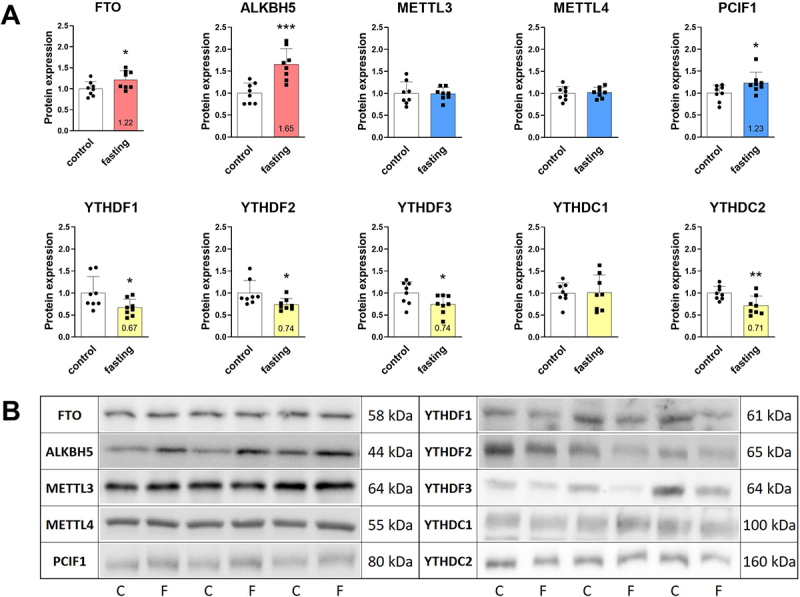
Effect of 3-day fasting on protein levels of m^6^A and m^6^Am regulators in the left ventricles assessed by Western blot (A). Writers are displayed in blue, erasers in red, and readers in yellow. The average of the control values is set to 1. Representative Western blot membranes (B). Protein loadings were 40 μg (YTHDF1, YTHDF3), 30 μg (YTHDC1), 20 μg (FTO, ALKBH5, YTHDC2), 15 μg (METTL3, YTHDF2), and 10 μg (METTL4, PCIF1). Values are means ± SD; *n* = 8; *p < 0.05; **p < 0.01; ***p < 0.001 (t-test). ALKBH5 – AlkB family member 5; C – control; F – fasting; FTO – fat mass and obesity-associated protein; METTL3 – methyltransferase-like 3; METTL4 – methyltransferase-like 4; PCIF1 – phosphorylated CTD interacting factor 1; YTHDF1–3 – YTH domain-containing family protein 1–3; YTHDC1–2 – YTH domain-containing protein 1–2.

Secondly, the protein levels of the main m^6^A and m^6^Am regulators were measured in LV samples using Western blot (Figure 3). Both erasers were up-regulated, FTO by 22%, and ALKBH5 by 65%, which corresponded to the transcript changes. Regarding writers, only m^6^Am methyltransferase PCIF1 was increased by 23%. Readers YTHDF1, YTHDF2, YTHDF3, and YTHDC2 were down-regulated by 33%, 26%, 26%, and 29%, respectively. Levels of other regulators (METTL3, METTL4, YTHDC1) were not affected on protein level.

Lastly, the peptide levels of m^6^A and m^6^Am regulators (2 peptides analysed for each protein) were also assessed using targeted proteomic analysis (Table 3). Out of the two peptides measured for each protein, the peptide with more profound changes was mentioned in the text. Regarding the main regulators, peptide levels of all YTHDF readers were decreased: YTHDF1 by 25%, YTHDF2 by 34%, and YTHDF3 by 27%. Demethylase ALKBH5, methyltransferase PCIF1, and reader YTHDC2 did not change significantly, but an increasing (ALKBH5, PCIF1) and decreasing (YTHDC2) trend was evident. FTO, METTL3, and YTHDC1 were unchanged at peptide levels. In addition to the main regulators, proteomic analysis revealed significant down-regulation also in peptide levels of other important proteins belonging to m^6^A machinery: methyltransferase METTL5 (by 50%); readers eIF3a (by 37%), eIF3g (by 26%), eIF3c (by 23%), and RBMX (by 16%); and repelled proteins USP10 (by 29%), CAPRIN1 (by 28%), G3BP2 (by 26%), G3BP1 (by 23%), and ELAVL1 (by 21%).

**Table 3.** Targeted proteomic analysis – changes in peptide levels.

Changes in peptide levels of epitranscriptomic regulators				
Protein	Protein Accession	Peptide	Change	P-value
**Writers**				
METTL5	B0BNB3	LFDTVIMNPPFGTK	−50%	0.004
		YDLPALYNFHK	−41%	0.038
WTAP	D3ZPY0	TTSSEPVDQAEATSK	−13%	0.076 ×
**Erasers**				
ALKBH5	D3ZKD3	YFFGEGYTYGAQLQK	67%	0.060 ×
**Readers**				
eIF3a	Q1JU68	ALEVIKPAHILQEK	−37%	0.049
YTHDF2	E9PU11	LGSTEVASSVPK	−34%	0.003
		APGMNTIDQGMAALK	−30%	0.021
eIF3a	Q1JU68	LLDMDGIIVEK	−28%	0.003
YTHDF3	D3ZIY3	AITDGQAGFGNDTLSK	−27%	0.002
eIF3g	Q5RK09	GFAFISFHR	−26%	0.009
YTHDF3	D3ZIY3	HTTSIFDDFAHYEK	−26%	0.012
YTHDF1	Q4V8J6	HTTSIFDDFSHYEK	−25%	0.013
eIF3c	B5DFC8	LNEILQVR	−23%	0.011
RBMX	Q4V898	GGHMDDGGYSMNFTLSSSR	−16%	0.025
eIF3g	Q5RK09	LPGELEPVQAAQNK	−17%	0.086 ×
**m^6^A-repelled proteins**				
USP10	Q3KR59	QADFVQTPITGIFGGHIR	−29%	0.021
CAPRIN1	Q5M9G3	TVLELQYVLDK	−28%	0.005
G3BP2	Q6AY21	VDAKPEVQSQPPR	−26%	0.007
CAPRIN1	Q5M9G3	YQEVTNNLEFAK	−24%	0.039
G3BP1	D3ZYS7	DFFQSYGNVVELR	−23%	0.023
ELAVL1	B5DF91	VAGHSLGYGFVNYVTAK	−21%	0.033

Changes at the edge of significance are marked by ’×’. ALKBH5 – AlkB family member 5; CAPRIN1 – Cell cycle associated protein 1; eIF3a/c/g – Eukaryotic initiation factor 3a/c/g; ELAVL1 – ELAV-like protein 1; G3BP1 – G3BP stress granule assembly factor 1; G3BP2 – G3BP stress granule assembly factor 2; RBMX – RNA-binding motif protein, X chromosome; USP10 – Ubiquitin specific peptidase 10; WTAP – Willms’ tumour 1-associating protein; YTHDC1 – YTH domain-containing protein 1; YTHDF1–3 – YTH domain-containing family protein 1–3.

**Figure 4.**
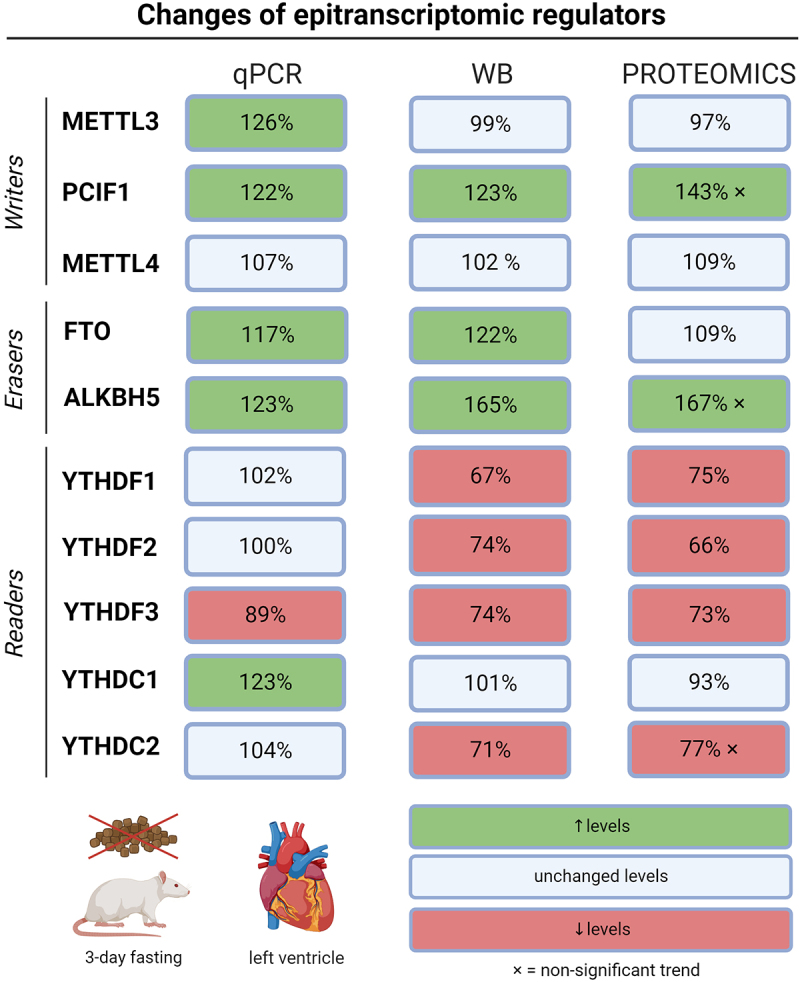
Levels of epitranscriptomic regulators in left ventricles of fasting rats assessed by RT-qPCR, Western blot, and proteomic analysis. Out of the two peptides measured for each protein in proteomic analyses, the peptide with more profound changes was depicted. ALKBH5 – AlkB family member 5; FTO – fat mass and obesity-associated protein; METTL3 – methyltransferase-like 3; METTL4 – methyltransferase-like 4; PCIF1 – phosphorylated CTD interacting factor 1; YTHDF1–3 – YTH domain-containing family protein 1–3; YTHDC1–2 – YTH domain-containing protein 1–2.

Despite the slight differences between the data from the three distinct methods (Figure 4), our results revealed that epitranscriptomic machinery was regulated in LV of rats subjected to 3-day fasting. A different gene expression regulation on transcriptional and translational levels can explain the minor discrepancies between transcript and protein levels.

### Effect of fasting on m^6^A/m levels in the left ventricles

**Figure 5.**
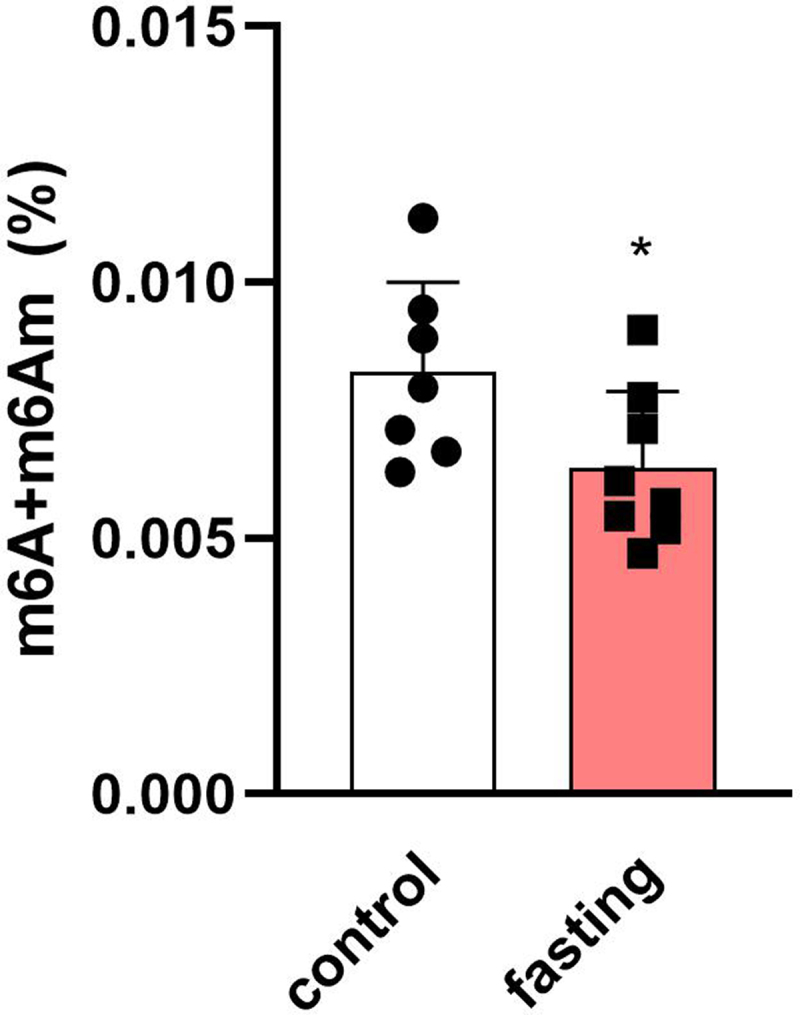
The difference between m^6^A/m methylation levels in total RNA from left ventricles of fasting rats. Values are means ± SD; *n* = 7–8; * *p* < 0.05 (t-test).

The effect of fasting on m^6^A/m methylation was evaluated in LV samples of fasting and control rats (Figure 5). The methylation levels were significantly decreased from 0.008% of total RNA to 0.006% of total RNA, which corresponded with increased protein levels of demethylases in fasting animals. These results demonstrated the cardiac epitranscriptomic regulation in the fasting heart.

### Methylation status of transcripts associated with cytoprotective functions of ketone bodies

**Figure 6.**
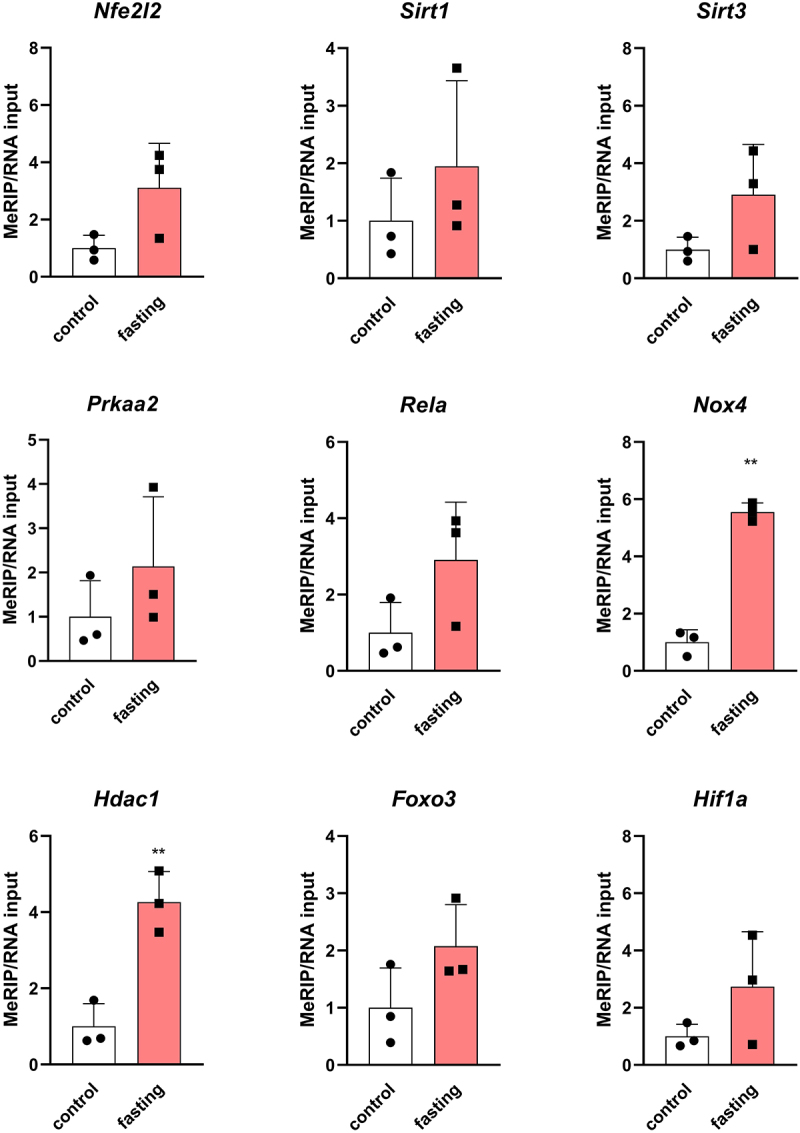
The m^6^A/m enrichment in specific mRnas isolated from left ventricles of fasting rats. The average of the control values is set to 1. Values are means ± SD; *n* = 3; ** *p* < 0.01 (t-test). *Nfe2l2* – NFE2 like BZIP transcription factor 2; *Sirt1* – sirtuin 1; *Sirt3* – sirtuin 3; *Prkaa2* – protein kinase AMP-activated catalytic subunit alpha 2; *Rela* – RELA proto-oncogene, NF-KB Subunit; *Nox4* – NADPH oxidase 4; *Hdac1* – histone deacetylase 1; *Foxo3* – forkhead box O3; *Hif1a* – hypoxia inducible factor 1 subunit alpha.

Ketolysis is the hallmark of fasting. We assessed the methylation levels in cardiac transcripts potentially associated with cell-protective functions of ketone bodies (Figure 6). Out of 9 selected transcripts, we found a 5-fold up-methylation of *Nox4* and 4-fold up-methylation of *Hdac1*. Other transcripts (*Nfe2l2*, *Sirt1*, *Sirt3*, *Prkaa2*, *Rela*, *Foxo3*, *Hif1a*) did not differ significantly between the control and fasting groups. However, upward trends were also obvious. These results showed altered epitranscriptomic regulations in possible cytoprotective pathways induced by fasting.

### Effect of FTO and ALKBH5 inhibition on AVCMs exposed to hypoxia

**Figure 7.**
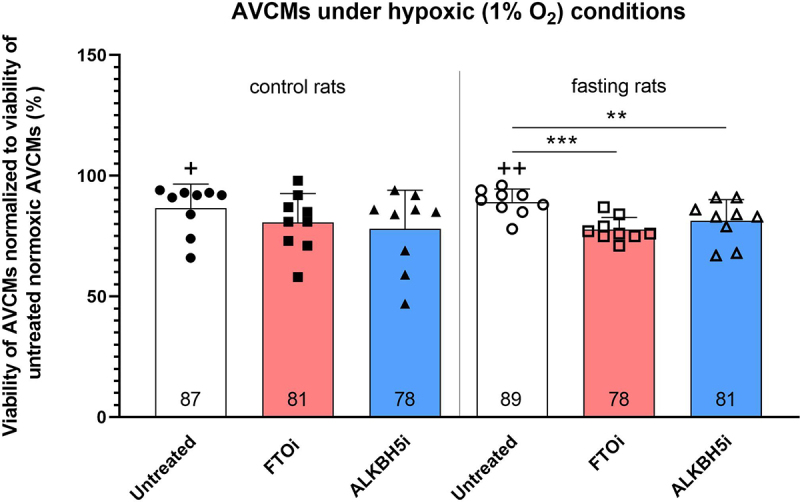
Effect of ALKBH5 and FTO inhibition on hypoxic tolerance of AVCMs isolated from control and fasting rats. Values are means ± SD; *n* = 9; ** *p* < 0.01; *** *p* < 0.001 (one-way ANOVA); + p < 0.05 compared to normoxic untreated AVCMs; ++ *p* < 0.01 compared to normoxic untreated AVCMs. ALKBH5i – ALKBH5 inhibitor; AVCMs – adult rat left ventricular cardiomyocytes; FTOi – FTO inhibitor.

To study the role of m^6^A and m^6^Am demethylases in the hypoxic tolerance of AVCMs, we examined the effect of FTO and ALKBH5 inhibitors on the viability of AVCMs from fasting and control rats using the SYTOX staining (Figure 7).

Administration of inhibitors did not affect the viability of AVCMs under normoxic conditions. Hypoxia significantly decreased the viability in untreated cells isolated from control and fasting rats to 87% and 89%, respectively. Under hypoxic conditions, inhibition of each demethylase further significantly decreased the viability of the cells from fasting rats to 81% (ALKBH5i) and 78% (FTOi), whereas the decrease in viability of cells from control rats did not reach statistical significance.

## Discussion

Our data indicate that the cardioprotective regime of 3-day fasting is associated with cardiac regulation of m^6^A and m^6^Am machinery. Out of the 33 epitranscriptomic regulators studied, 22 were affected by fasting on either mRNA or protein levels, including up-regulation of demethylases FTO and ALKBH5. Together with that, we also observed decreased m^6^A/m methylation in the hearts of fasting animals. Fasting was also associated with the up-methylation of *Nox4* and *Hdac1* transcripts, potentially participating in cell-protective functions of ketone bodies produced during fasting [31]. Inhibition of either demethylase resulted in decreased hypoxic tolerance in AVCMs isolated from fasting animals. Hence, we suggest that epitranscriptomics might act as an important layer of gene expression regulation in the fasting heart and contribute to cardioprotection.

## Fasting affects cardiac epitranscriptomic regulations

Epitranscriptomic modification m^6^A and its main regulators are affected in the heart under various physiological and pathophysiological conditions [37]. However, the role of m^6^A and m^6^Am in cardioprotection and cardioprotective models remains poorly described. In the past, the demethylases FTO and ALKBH5 were mostly associated with cardioprotective effects [18–20]. We also previously showed that the cardioprotective 4-week adaptation of rats to chronic hypoxia increased the protein expression of these demethylases [38]. However, the participation of epitranscriptomic regulations in fasting, another cardioprotective model, was still hypothetical. Recently, Xu et al. [22] discovered that intermittent fasting (IF) protects the mouse heart via a mechanism associated with decreased m^6^A-RNA methylation levels. In line with this, they found that METTL3 levels were down-regulated and FTO levels were up-regulated after IF intervention. In our experiments, we found decreased methylation levels after 3 days of fasting as well. Regarding FTO, we observed an increase in its expression by RT-qPCR and Western blot techniques; however, the proteomic analysis did not confirm significant up-regulation. The discrepancy appeared in the case of METTL3, where we found increased gene expression by RT-qPCR and no changes in protein levels. This might be explained by the different fasting models (fasting every other day for 8 weeks vs. ‘short-term’ fasting for 3 days) and also different animal models used (mice vs. rats). Regarding the other main regulators not analysed by Xu et al., we observed up-regulation of the second demethylase ALKBH5 and methyltransferase PCIF1 at both gene and protein levels. Gene expression of reader *Ythdc1* was also increased. Other readers (YTHDF1–3 and YTHDC2) decreased their protein levels, and only *Ythdf3* was down-regulated on the gene level.

In addition to the main m^6^A and m^6^Am machinery, our study analysed less-known regulators in fasting hearts by targeted proteomic analysis. Their role in the fasting heart is unclear, but important functions in cardiac biology were suggested for these proteins. The most down-regulated protein was methyltransferase METTL5. This m^6^A writer has been shown to regulate mRNA translation via 18S rRNA methylation [39]. Cardiac-specific depletion of METTL5 promoted pressure overload-induced cardiomyocyte hypertrophy and adverse remodelling [40]. Other affected proteins, which were down-regulated, included eIF3 reader subunits (eIF3a/c/g) and reader RBMX. eIF3 is a key factor in translation regulation. The largest and most well-known member of the eIF family is eIF3a. A mutation in the *eIF3a* gene was uncovered in patients with left ventricular non-compaction cardiomyopathy, a hereditary disease manifested by thromboembolic complications, arrhythmias, and heart failure. Further analyses on H9c2 cells showed that this mutation was associated with decreased proliferation and induction of apoptosis [41]. The role of *eIF3a* was also described in cardiac fibrosis [42]. Another eIF3 subunit, eIF3c, was identified as a direct target of the reader YTHDF1, which augmented the translation of eIF3c in an m^6^A-dependent manner [43]. In fasting hearts, we observed down-regulation of the YTHDF1-eIF3c axis as levels of both regulators were decreased. In addition to m^6^A readers, the majority of m^6^A-repelled proteins were less expressed in fasting hearts (G3BP1/2, ELAVL1, USP10, CAPRIN1). G3BP1 was found to be an important regulator of cardiac hypertrophy, atrial fibrillation, and coronary heart disease [44]. G3BP2 was also involved in the induction of cardiac hypertrophy and contributed to the development of atherosclerosis [45–47]. Moreover, overexpression of G3BP2 partially reversed the hypoxia/reoxygenation (H/R)-induced apoptosis in H9c2 cells [48]. However, in the cardioprotective fasting model, we found decreased G3BP2 levels. ELAVL1 is another RNA-binding protein with diverse cellular roles. Among other functions, it associates with mRNAs encoding hypoxia-response proteins such as hypoxia-inducible factor 1α (HIF-1α) or vascular endothelial growth factor (VEGF) and enhances their expression after hypoxia [49]. Myocardial I/R injury was linked with the up-regulation of ELAVL1 level [50]. Knockdown of ELAVL1 reduced MI-induced cardiomyocyte apoptosis, infarct size, and fibrosis area [50,51]. Therefore, decreased ELAVL1 levels observed in fasting hearts could play a role in the induction of cardioprotective phenotype. Protein USP10 was associated with cardiac hypertrophy [52,53]. Also, levels of this m^6^A-repelled protein were decreased in H9c2 cells after the H/R insult, and overexpression of USP10 increased the viability and suppressed the apoptosis of H/R-induced cells [54]. However, this regulator was down-regulated in fasting hearts.

### *Fasting is associated with up-methylation of* Nox4 *and* Hdac1 *transcripts in the heart*

Given the association between fasting and ketolysis, our study focused on transcripts potentially related to the cytoprotective functions of ketone bodies, examining their m^6^A/m methylation levels. We found that *Nox4* was significantly up-methylated in fasting hearts. NOX4 generates reactive oxygen species, which are involved in various signalling pathways including cardiac adaptation to different types of physiological and pathophysiological stresses. The protective role of NOX4 in the heart has been described [55]. However, ROS production by the NOX4 enzyme may also be harmful to the heart since ROS are a double-edged sword. It has been reported that NOX4 underwent extensive alternative splicing in human hearts and that the full-length NOX4 was significantly up-regulated in ischaemic cardiomyopathy [56]. As splicing regulation is one of the primary functions of m^6^A and m^6^Am modifications [8,14], alterations in methylation levels in the *Nox4* transcript may be crucial for determining the heart’s fate. Besides *Nox4*, we also detected *Hdac1* up-methylation after fasting. HDAC1 functions as an epigenetic regulator by removing acetyl groups from histones and is inhibited by 3-hydroxybutyrate [31], the main ketone body increased in our fasting model. Inhibition of this protein was also associated with protecting cardiomyocytes against hypoxia [57]. Thus, these results revealed altered epitranscriptomic regulation in cell-protective pathways induced by ketone bodies in fasting hearts.

### Inhibition of demethylases decreases the hypoxic tolerance of AVCMs isolated from fasting rats

The changes observed in fasting hearts included up-regulation of both demethylases on both transcript and protein levels. We did not observe a significant effect of FTO inhibition or ALKBH5 inhibition on hypoxic tolerance (1% O_2_, 24 h) in cells from control animals, even though the decreasing trend was evident. However, a significant reduction of AVCM viability appeared in the fasting group after FTOi and ALKBH5i treatment.

It was already reported that FTO affects the survival of cardiomyocytes subjected to H/R. FTO was poorly expressed in human cardiomyocyte cell line AC16 exposed to H/R while FTO up-regulation improved the viability after H/R insult [18]. Similarly, in mouse cardiomyocytes, FTO overexpression inhibited apoptosis induced by acute H/R, while FTO knockdown had the opposite effect [19]. Moreover, the downexpression of FTO was observed in mouse hearts and isolated mouse cardiomyocytes subjected to I/R and acute H/R insults, respectively. FTO overexpression then attenuated the H/R-induced apoptosis in these cells [20]. Similarly, ALKBH5 overexpression also inhibited apoptosis of H/R-treated cardiomyocytes [15]. In line with these observations, our data confirm that the activity of RNA demethylases FTO and ALKBH5 is important for cardiomyocyte tolerance to hypoxic insult.

## Conclusion

This study revealed that the cardioprotective regime of fasting altered m^6^A/m modifications and its regulators in the heart, including demethylases FTO and ALKBH5, which were up-regulated after fasting. Specific transcripts potentially associated with the cell-protective functions of ketone bodies induced by fasting showed differential methylation in fasting hearts. Moreover, the inhibition of demethylases FTO and ALKBH5 decreased the hypoxic tolerance of cardiomyocytes isolated from fasting rats. In summary, these results suggest that epitranscriptomic regulations participate in the induction of cardioprotective phenotype induced by fasting.

## Supplementary Material

Supplements Benak.docx
