# Supplementary material for: Epitranscriptomic regulation in fasting hearts: implications for cardiac health
Source: RNA Biol. 2024 Feb 7;21(1):1–14. doi: 10.1080/15476286.2024.2307732 (PMC10854364; doi:10.1080/15476286.2024.2307732)
Supplement: Supplements Benak.docx [file KRNB_A_2307732_SM1806.docx]

# Supplementary materials

## Materials and Methods

**Lipidomics and metabolomics**

Global lipidomic and metabolomic profiling of plasma samples was conducted using a combined untargeted and targeted workflow for the lipidome, metabolome, and exposome analysis (LIMeX) [1-3] with some modifications. Extraction was carried out using a biphasic solvent system of cold methanol, methyl tert-butyl ether (MTBE), and 10% methanol. Four different liquid chromatography-mass spectrometry (LC-MS) platforms were used for profiling: (i) lipidomics of complex lipids using reversed-phase liquid chromatography with mass spectrometry (RPLC-MS) in positive ion mode, (ii) lipidomics of complex lipids in RPLC-MS in negative ion mode, (iii) metabolomics of polar metabolites using hydrophilic interaction chromatography with mass spectrometry (HILIC-MS) in positive ion mode, and (iv) metabolomics of polar metabolites using RPLC-MS in negative ion mode.

**Sample extraction for metabolomics and lipidomic analyses**

Plasma samples (25 µL) were mixed with 765 μL of cold methanol/MTBE mixture (165 µL + 600 µL, respectively) containing internal standards and shaken for 30 s. Then, 165 µL of 10% MeOH with internal standards was added, shaken for 30 s, and centrifuged at 16,000 rpm for 5 min.

For lipidomic profiling, 100 µL of the upper organic phase was collected, evaporated, and resuspended using 100 µL methanol with internal standard (12-[[(cyclohexylamino)carbonyl]amino]- dodecanoic acid, CUDA), shaken for 30 s, and centrifuged at 16,000 rpm for 5 min and used for LCMS analysis.

For metabolomic profiling, 70 µL of the bottom aqueous phase was collected, evaporated, resuspended in 70 µL of an acetonitrile/water (4:1, v/v) mixture with internal standards (CUDA and Val-Tyr-Val), shaken for 30 s, and centrifuged at 16,000 rpm for 5 min and analyzed using HILIC metabolomics platform. Another 70 µL aliquote of bottom aqueous phase was mixed with 210 µL of an isopropanol/acetonitrile (1:1, v/v) mixture, shaken for 30 s, centrifuged at 16,000 rpm for 5 min, and the supernatant was evaporated, resuspended in 5% methanol/0.2% formic acid with internal standards (CUDA and Val-Tyr-Val), shaken for 30 s, centrifuged at 16,000 rpm for 5 min and analyzed using RPLC metabolomics platform.

**LC-MS-based lipidomics**

The LC-MS systems consisted of a Vanquish UHPLC System (Thermo Fisher Scientific, Waltham, MA, USA) coupled to a Q Exactive Plus mass spectrometer (Thermo Fisher Scientific, Waltham, MA, USA).

Lipids were separated on an ACQUITY Premier BEH C18 column (50 × 2.1 mm; 1.7 μm) coupled to a VanGuard FIT cartridge (5 × 2.1 mm; 1.7 μm) (Waters, Milford, MA, USA). The column was maintained at 65°C at a flow rate of 0.6 mL/min. For LC–ESI(+)-MS analysis, the mobile phase consisted of (A) 60:40 (v/v) acetonitrile:water with ammonium formate (10 mM) and formic acid (0.1%) and (B) 90:10:0.1 (v/v/v) isopropanol:acetonitrile:water with ammonium formate (10 mM) and formic acid (0.1%). For LC–ESI(−)-MS analysis, the composition of the solvent mixtures was the same except for the addition of ammonium acetate (10 mM) and acetic acid (0.1%) as mobile-phase modifiers. Separation was conducted under the following gradient for LC–ESI(+)-MS: 0 min 15% (B); 0–1 min 30% (B); 1– 1.3 min from 30% to 48% (B); 1.3–5.5 min from 48% to 82% (B); 5.5–5.8 min from 82% to 99% (B); 5.8–6 min 99% (B); 6–6.1 min from 99% to 15% (B); 6.1–7.5 min 15% (B). For LC–ESI(−)- MS, the following gradient was used: 0 min 15% (B); 0–1 min 30% (B); 1–1.3 min from 30% to 48% (B); 1.3–4.8 min from 48% to 76% (B); 4.8–4.9 min from 76% to 99% (B); 4.9–5.3 min 99% (B); 5.3–5.4 min from 99% to 15% (B); 5.4–6.8 min 15% (B). The injection volumes were 2 μL in ESI(+) and 5 µL in ESI(−). Sample temperature was maintained at 4°C.

The ESI source and MS parameters were: sheath gas pressure, 60 arbitrary units; aux gas flow, 25 arbitrary units; sweep gas flow, 2 arbitrary units; capillary temperature, 300°C; aux gas heater temperature, 370°C; MS1 mass range, *m*/*z* 200–1700; MS1 resolving power, 35,000 FWHM (m/z 200); number of data-dependent scans per cycle, 3; MS/MS resolving power, 17,500 FWHM (m/z 200). For ESI(+), a spray voltage of 3.6 kV and normalized collision energy of 20% was used; for ESI(−), a spray voltage of −3.0 kV and normalized collision energy of 10, 20 and 30% were set up.

**LC-MS-based metabolomics**

Polar metabolites were separated on an ACQUITY Premier BEH Amide column (50 × 2.1 mm; 1.7 μm) coupled to a VanGuard FIT cartridge (5 × 2.1 mm; 1.7 μm) (Waters, Milford, MA, USA). The column was maintained at 45°C at a flow rate of 0.4 mL/min. The mobile phase consisted of (A) water with ammonium formate (10 mM) and formic acid (0.125%) and (B) acetonitrile:water (95/5) with ammonium formate (10 mM) and formic acid (0.125%). Separation was conducted under the following gradient: 0 min 100% (B); 0–1 min 100% (B); 1–3.9 min from 100% to 70% (B); 3.9–5.1 min from 70% to 30% (B); 5.1–6.4 min from 30% to 100%(B); 6.4– 8.0 min 100% (B). The injection volume of 0.6 µL was used. Sample temperature was maintained at 4°C.

Polar metabolites were also separated on an ACQUITY Premier HSS T3 column (50 × 2.1 mm; 1.8 μm) coupled to a VanGuard FIT cartridge (5 × 2.1 mm; 1.8 μm) (Waters, Milford, MA, USA). The column was maintained at 45°C using a ramped flow rate. The mobile phase consisted of (A) water with formic acid (0.2%) and (B) methanol with formic acid (0.1%). Separation was conducted under the following gradient: 0 min 1% (B) 0.3 mL/min; 0–0.5 min 1% (B) 0.3 mL/min; 0.5–2 min from 1% to 60% (B) 0.3 mL/min; 2–2.3 min from 60% to 95% (B) from 0.3 mL/min to 0.5 mL/min; 2.3–3.0 min 95% (B) 0.5 mL/min; 3.0–3.1 min from 95% to 1% (B) 0.5 mL/min; 3.1–4.5 min 1% (B) 0.5 mL/min; 4.5–4.6 min 1% (B) from 0.5 mL/min to 0.3 mL/min; 4.6–5.5 min 1% (B) 0.3 mL/min. An injection volume of 5 μL was used. Sample temperature was maintained at 4°C.

The ESI source and MS parameters were: sheath gas pressure, 50 arbitrary units; aux gas flow, 13 arbitrary units; sweep gas flow, 3 arbitrary units; capillary temperature, 260°C; aux gas heater temperature, 425°C; MS1 mass range, *m*/*z* 60–900; MS1 resolving power, 35,000 FWHM; number of data-dependent scans per cycle, 3; MS/MS resolving power, 17,500 FWHM. A spray voltage of 3.6 kV and −2.5 kV for ESI(+) and ESI(–), respectively, was used. A normalized collision energy of 20, 30 and 40% was used for all metabolomics platforms.

**Quality control**

Quality control was assured by (i) randomization of the actual samples within the sequence, (ii) injection of quality control (QC) pool samples at the beginning and the end of the sequence and between each 10 actual samples, (iii) analysis of procedure blanks, (iv) serial dilution of QC sample (0, 1/16, 1/8, 1/4, 1/2, 1), and (v) checking the peak shape and the intensity of spiked internal standards and the internal standard added prior to injection.

**Data processing**

Quality LC-MS data from metabolomic and lipidomic profiling were processed through MS-DIAL v. 4.70 software. Metabolites were annotated using in-house retention time–*m*/*z* library and MS/MS libraries from commercial and open sources (NIST20, MassBank, MoNA). Lipids were annotated using LipidBlast built in MS-DIAL. Raw data were filtered using blank samples (a max sample peak height/blank peak height average < 10), serial dilution samples (an *R*^2^ < 0.8), and QC pool samples with relative standard deviation (RSD) > 30%.

1. Sistilli, G., et al., *Krill Oil Supplementation Reduces Exacerbated Hepatic Steatosis Induced by Thermoneutral Housing in Mice with Diet-Induced Obesity.* Nutrients, 2021. **13**(2).

2. Janovska, P., et al., *Dysregulation of epicardial adipose tissue in cachexia due to heart failure: the role of natriuretic peptides and cardiolipin.* J Cachexia Sarcopenia Muscle, 2020. **11**(6): p. 1614-1627.

3. Tsugawa, H., et al., *A lipidome atlas in MS-DIAL 4.* Nat Biotechnol, 2020. **38**(10): p. 1159-1163.

## Tables

**Table S1.** TaqMan Gene Expression Assays

| **Gene** | **Catalog number** | **Assay ID** | **Specification** |
| --- | --- | --- | --- |
| *Mettl3* | 4331182 | Rn01414796_m1 | FAM-MGB |
| *Pcif1* | 4351372 | Rn01423448_m1 | FAM-MGB |
| *Mettl4* | 4351372 | Rn04244733_m1 | FAM-MGB |
| *Fto* | 4331182 | Rn01538186_m1 | FAM-MGB |
| *Alkbh5* | 4351372 | Rn01750503_m1 | FAM-MGB |
| *Ythdf1* | 4331182 | Rn00620538_m1 | FAM-MGB |
| *Ythdf2* | 4351372 | Rn01180761_m1 | FAM-MGB |
| *Ythdf3* | 4351372 | Rn01289124_m1 | FAM-MGB |
| *Ythdc1* | 4331182 | Rn00591592_m1 | FAM-MGB |
| *Ythdc2* | 4351372 | Rn01256278_m1 | FAM-MGB |
| *Hprt1* | 4331182 | Rn01527840_m1 | VIC-MGB_PL |
| *Nupl2* | 4331182 | Rn01442493_m1 | VIC-MGB_PL |
| *Sdha* | 4331182 | Rn00590475_m1 | VIC-MGB_PL |
| *Tomm22* | 4331182 | Rn01502295_g1 | VIC-MGB_PL |
| *Top1* | 4331182 | Rn00575128_m1 | VIC-MGB_PL |
| *Ywhaz* | 4448484 | Rn00755072_m1 | VIC-MGB_PL |

*Alkbh5* – AlkB family member 5; *Fto* – Fat mass and obesity-associated; *Hprt* – hypoxanthine phosphoribosyltransferase 1; *Mettl3* – Methyltransferase‑like 3; *Mettl4* – Methyltransferase-like 4; *Nupl2* – nucleoporin-like 2*; Pcif1* – Phosphorylated CTD interacting factor 1; *Sdha* – succinate dehydrogenase complex flavoprotein subunit A; *Tomm22* – translocase of outer mitochondrial membrane 22; *Top1* – topoisomerase I; *Ythdf1-3* – YTH domain-containing family protein 1-3; *Ythdc1-2* – YTH domain-containing protein 1-2; *Ywhaz* – tyrosin-3-monooxygenase/tryptophan 5 monooxygenase activation protein zeta.

**Table S2.** TaqMan Gene Expression Assays

| **Gene** | **Catalog number** | **Assay ID** | **Specification** |
| --- | --- | --- | --- |
| *Nfe2l2* | 4331182 | Rn00582415_m1 | FAM-MGB |
| *Sirt1* | 4331182 | Rn01428096_m1 | FAM-MGB |
| *Sirt3* | 4331182 | Rn01501410_m1 | FAM-MGB |
| *Prkaa2* | 4331182 | Rn00576935_m1 | FAM-MGB |
| *Rela* | 4331182 | Rn01502266_m1 | FAM-MGB |
| *Nox4* | 4331182 | Rn00585380_m1 | FAM-MGB |
| *Hdac1* | 4331182 | Rn01519308_g1 | FAM-MGB |
| *Foxo3* | 4331182 | Rn01441087_m1 | FAM-MGB |
| *Hif1a* | 4331182 | Rn01472831_m1 | FAM-MGB |

*Nfe2l2* – NFE2 like BZIP transcription factor 2; Sirt1 – Sirtuin 1; *Sirt3* – Sirtuin 3; *Prkaa2* – Protein kinase AMP-activated catalytic subunit alpha 2; *Rela* – RELA proto-oncogene, NF-KB Subunit; *Nox4* – NADPH oxidase 4; *Hdac1* – Histone deacetylase 1; *Foxo3* – forkhead box O3; *Hif1a* – Hypoxia inducible factor 1 subunit alpha.

## Figures

Fig S1: Effect of different inhibitor concentrations (FTOi and ALKBH5i) on the viability of AVCMs. Values are means ± S.D.; n = 3 – 10; * P < 0.05 vs. control group.
